# Systematic Surveillance of Rickettsial Diseases in 27 Hospitals from 26 Provinces throughout Vietnam

**DOI:** 10.3390/tropicalmed7060088

**Published:** 2022-05-31

**Authors:** Nguyen Vu Trung, Le Thi Hoi, Tran Mai Hoa, Dang Thi Huong, Ma Thi Huyen, Vuong Quang Tien, Dao Thi Tuyet Mai, Nguyen Thi Thu Ha, Nguyen Van Kinh, Christina M. Farris, Allen L. Richards

**Affiliations:** 1 Hanoi Medical University, Hanoi 100000, Vietnam; nguyenvutrung@hmu.edu.vn (N.V.T.); lethihoi@hmu.edu.vn (L.T.H.); maihoatran@hmu.edu.vn (T.M.H.); danghuong.yhdp.hmu@gmail.com (D.T.H.); mathihuyen@hmu.edu.vn (M.T.H.); vuongquangtien_t59@hus.edu.vn (V.Q.T.); mechelia.alba117@gmail.com (D.T.T.M.); 2 Pasteur Institute in Ho Chi Minh City, Ho Chi Minh City 700000, Vietnam; 3 Hanoi University of Public Health, Hanoi 100000, Vietnam; ntth1@huph.edu.vn; 4 National Hospital for Tropical Diseases, Hanoi 100000, Vietnam; kinhnv@nhtd.vn; 5 Viral and Rickettsial Diseases Department, Naval Medical Research Center, Silver Spring, MD 20910, USA; christyfarris@gmail.com; 6 Department of Preventive Medicine and Biostatistics, Uniformed Services University of the Health Sciences, Bethesda, MD 20814, USA

**Keywords:** rickettsial diseases, scrub typhus, murine typhus, spotted fever rickettsioses, hospital surveillance, epidemiology

## Abstract

In Vietnam, the public health burden of rickettsial infections continues to be underestimated due to knowledge gaps in the epidemiology of these diseases. We conducted a systematic study among 27 hospitals from 26 provinces in eight ecological regions throughout Vietnam to investigate the prevalence, distribution, and clinical characteristics of rickettsial diseases. We recruited 1834 patients in the study from April 2018 to October 2019. The findings showed that rickettsial diseases were common among undifferentiated febrile patients, with 564 (30.8%) patients positive by qPCR for scrub typhus, murine typhus or spotted fever. Scrub typhus (484, 85.8%) was the most common rickettsial disease, followed by murine typhus (67, 11.9%) and spotted fever (10, 1.8%). Rickettsial diseases were widely distributed in all regions of Vietnam and presented with nonspecific clinical manifestations.

## 1. Introduction

Rickettsial diseases are caused by infection with obligate intracellular bacteria of two genera, *Rickettsia* and *Orientia*, of the family Rickettsiaceae, in the order Rickettsiales. These pathogens are transmitted to humans by arthropod vectors such as mites, ticks, fleas and lice. Rickettsial agents can be divided into three groups based upon genotyping and antigenicity: typhus group rickettsiae (TGR), spotted fever group rickettsiae (SFGR) and scrub typhus group orientiae (STGO) [1,2]. Additionally there is a transition group of rickettsiae which diverge from SFGR genetically, but remain in the SFGR antigenic group. For the purposes of this report, the transition group rickettsiae are grouped with the SFGR. Rickettsial species cause acute and endemic diseases in many parts of the world, especially in Asia [3,4,5,6]. The clinical manifestations of rickettsial diseases vary widely, ranging from mild to life-threatening illness depending upon the rickettsial species or strain, underlying patient diseases, and delay in time to appropriate medical care. Rickettsial diseases usually present with acute fever, headache, malaise, a possible rash (usually maculopapular), and/or an eschar (a lesion at the bite site of the infected arthropod vector). Since the target cell for the rickettsial infection is the endothelial cell, all organs and tissues may be infected and therefore clinical manifestations of the disease can involve various organs, including the liver, lungs, kidneys, and heart [7,8,9].

In Vietnam, knowledge about the prevalence, incidence and distribution of rickettsial diseases is limited. This lack of knowledge combined with misconceptions and outdated clinical guidelines leads to the under diagnosis of these diseases in clinical practice at district, provincial and even national hospitals [10,11]. The presence of scrub typhus was described in Saigon (Ho Chi Minh City) as early as 1915 [12] and scrub typhus and murine typhus were frequently reported among American servicemen stationed in southern Vietnam during the Vietnam war [13,14,15]. However, to date there have been no systematic investigations of scrub typhus, murine typhus and other rickettsial diseases in many parts of the country. Recently, a few reports found that rickettsial diseases were common causes of hospital admissions in Northern and Central Vietnam. While these investigations were limited in terms of the number of patients, the findings were noteworthy as to the number of patients that met the clinical criteria for testing for scrub typhus and murine typhus that were positive: 34.1–43.8% and 3.0–33.3%, respectively [9,16,17]. Of the three groups of rickettsial diseases, the presence and distribution of SFGR infections in Vietnam is least recognized. In 2015, a serologic survey for antibodies against rickettsiae in healthy individuals in urban and rural districts in Northern Vietnam (Hanoi) found that the overall antibody prevalence of SFGR was 1.7% [18]. More recently, molecular detection of *Rickettsia felis*, an SFGR, in two patients with acute undifferentiated fevers and one control in a prospective case-control study conducted in four hospitals in central Vietnam (Quang Nam province) provided the first evidence of human *R. felis* infection in Vietnam [11].

To address the gap in knowledge of rickettsial infections, a systematic study involving 27 national/provincial hospitals in 26 provinces throughout Vietnam was conducted to investigate the prevalence, distribution, and clinical characteristics of rickettsial diseases. This study has not only provided a comprehensive view of the prevalence and distribution of these infections within Vietnam, but it has also strengthened clinical diagnostic and laboratory capacity for the diagnosis of rickettsial diseases.

## 2. Materials and Methods

### 2.1. Study Design

A prospective observational hospital-based investigation was conducted in five national and 22 provincial hospitals among 26 provinces throughout Vietnam from April 2018 to October 2019. The provinces represent all eight agroecological regions of Vietnam and were selected according to the following criteria: (1) provinces/cities with national or referral hospitals were prioritized (Hanoi, Ho Chi Minh City, Thua Thien-Hue, Thai Nguyen, Can Tho); (2) the number of selected provinces accounted for one third to two thirds of the total provinces in each agroecological region; (3) non-neighboring provinces within an agroecological region took precedence over neighboring provinces when possible.

Patients with clinically suspected rickettsiosis who met at least one of three inclusion criteria were recruited into the study: (1) had a fever and an eschar; (2) had a fever and at least one of following clinical signs or symptoms: rash, headache, myalgia, conjunctivitis, swollen lymph nodes, hepatomegaly, or splenomegaly and had no other cause of fever identified after available laboratory tests such as dengue IgM/IgG/NS1, malaria rapid diagnostic test/staining of malaria parasites, measles IgM rapid diagnostic test, influenza A/B rapid test, rubella IgG/IgM rapid test, blood culture were performed; or (3) had a fever and was in a severe/life-threatening condition or was suspected of having rickettsial disease. Patients were excluded from the study if they had: (1) tested positive for malaria, dengue fever, measles, flu, and/or rubella or had clinical presentations, laboratory test results, X-ray images of pneumonia, sepsis, urinary tract infection or other infections caused by identified bacterial pathogens; (2) used antibiotics for the treatment of rickettsial diseases such as chloramphenicol, doxycycline and azithromycin for more than two days; or (3) did not agree to participate in the study.

Patient information including demographics, medical history, clinical presentations, laboratory results at four time points (admission to the hospital, enrollment in the study, 3-day post enrollment, and discharge), treatment regimens and outcomes were collected in case record forms (CRF).

### 2.2. Specimen Collection and Processing

Upon enrollment in the study, 4 mL of whole blood was collected from each patient in K2-EDTA tubes and sent to the laboratory of each hospital for peripheral blood mononuclear cell (PBMC) isolation by density gradient. Briefly, the whole blood was added to 2 mL of Ficoll-Paque PLUS (GE Healthcare, Chicago, IL, USA) in a Corning^®^ 15 mL centrifuge tube and centrifuged at 1800× *g* for 15 min. The plasma layer was removed and the PBMCs were transferred to a cryotube. The PBMCs were stored at −80 or −20 °C until being transported to the National Hospital for Tropical Diseases and Hanoi Medical University, Hanoi for further analysis.

#### 2.2.1. DNA Extraction

PBMC samples were manually processed using QIAamp DNA Mini Kits, Qiagen (Hilden, Germany) according to the manufacturer’s instructions. For each patient, 200 μL of PBMCs were used for DNA extraction. Extracted DNA from each specimen was eluted into 120 μL of Qiagen AE buffer. DNA was stored at 4 °C for testing within a week or −80 °C for later testing.

#### 2.2.2. Quantitative Real-Time PCR Assays

Nucleic acid from *Rickettsia* species was detected in DNA preparations by a genus-specific quantitative real-time PCR (qPCR) assay (Rick17b) as previously described [19,20]. Samples found to be positive by the Rick17b qPCR assay were further tested for the presence of *R. typhi* and *R. prowazekii* DNA using the *R. typhi* species-specific qPCR assay (Rtyph) and the *R. prowazekii* specific assay (Rprow), which target portions of the outer membrane protein B gene (*ompB*) of each pathogen [19,20].

*Orientia tsutsugamushi* DNA was detected using the Otsu47 qPCR assay, which targets the 47 kDA HtrA gene (*htrA*) [21,22].

#### 2.2.3. Multilocus Sequence Typing (MLST) for Rickettsia Species Identification

Patient DNA samples that screened positive with the Rick17b genus-specific qPCR assay and negative with the Rtyphi and Rprow qPCR assays were subjected to the MLST scheme described by Fournier et al. [23] to determine the *Rickettsia* species present in the sample. The MLST scheme utilized five genes that encode the conserved proteins of the 17 kDa antigen and the key tricarboxylic acid cycle enzyme, citrate synthase (*gltA*), as well as the more variable outer membrane protein (Omp)A, OmpB, and surface cell antigen four (Sca4). Through sequencing, results from previous studies have confirmed phylogenetic distinctness for the species and subspecies of *Rickettsia* utilizing this procedure [21,23,24]. Unfortunately, amplicons were not obtained from ten DNA samples that were positive by Rick17b (Ct values ranged from 35 to 38) and negative by Rtyphi and Rprow and therefore species identity was not achieved.

### 2.3. Data Entry and Analysis

The patient information detailed in the case report forms was entered into Epidata 3.2 (The EpiData Association, Odense, Denmark). To minimize data entry errors, double data entry was performed by two different people. Data was then analyzed using STATA 12.0 (StataCorp, College Station, TX, USA). Categorical variables were summarized as frequencies and percentages. Non-normally distributed variables were described as medians with interquartile ranges (IQR). The Fisher’s exact and Mann-Whitney tests were used for the comparison of the proportion and mean between groups, respectively. Distribution maps were created using ArcMap 10.2 (Esri, Redlands, CA, USA).

### 2.4. Ethical Considerations

Written informed consent was obtained from patients ≥18 years of age. For patients aged 12–17 years, consent was obtained from both patients and their parents/guardians. Parents/guardians of patients less than 12 years of age provided consent. The study protocol was approved by National Hospital for Tropical Diseases, Hanoi Medical University, and Naval Medical Research Center Institutional Review Boards in compliance with all applicable federal regulations governing the protection of human subjects (NHTD 24/HĐĐĐ-NĐTƯ, HMU 129/GCN-HĐĐĐNCYSH-ĐHYHN, NMRC PJT 18-01).

## 3. Results

### Patient Population and Enrollment

**Figure 1:**
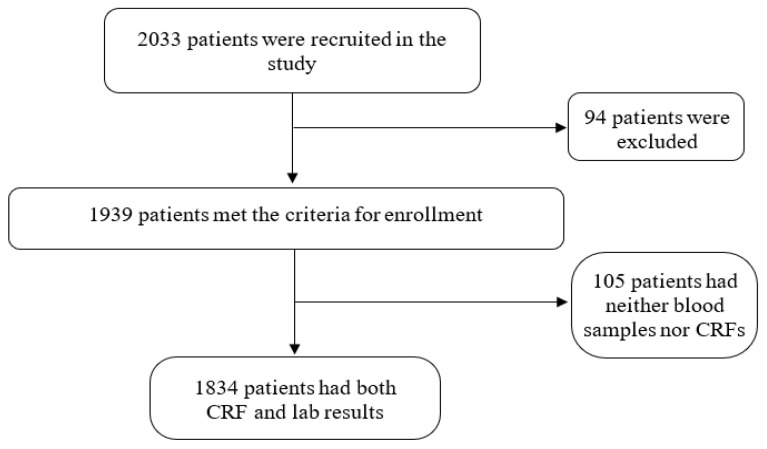
Enrollment flow of the study.

Between April 2018 and October 2019, 2033 patients were recruited at 27 hospitals among 26 provinces. Of those, 94 patients were excluded from the study resulting in a total of 1939 enrolled patients. The most common reasons for exclusion were: (1) pre-enrollment use of antibiotics (i.e., chloramphenicol, doxycycline or azithromycin) for more than two days (28 patients); (2) a positive blood culture (i.e., *Escherichia coli*, *Klebsiella pneumoniae*, *Staphylococcus aureus*, *Streptococcus suis*, *Salmonella* sp., *Burkholderia cepacia*, *Acinetobacter baumannii*) (16 patients); and (3) a positive laboratory test results for another infectious disease such as dengue fever, flu, measles, malaria, HIV (12 patients). Additionally, one patient was excluded based on a systemic lupus erythematosus diagnosis and four were excluded for living outside Vietnam (i.e., Cambodia or Laos) at the time of symptom onset. Patients were also excluded for failing to adhere to the inclusion criteria or for not having blood samples collected on the day of admission for testing. Of the 1939 enrolled patients, 105 had either no case report forms or no blood samples due to storage problems and therefore were subsequently disenrolled. Thus, the final study population was 1834 (Figure 1).

Table 1 shows the general characteristics of patients enrolled in the study as well as the comparison of qPCR negative with positive groups. The median age of the study population was 39 years (range 27–55 years). More than two-thirds (1271; 69.4%) of patients lived in rural areas and farmer was the most common occupation (861, 47.0%). Close to a quarter of all patients (426, 23.2%) had used antibiotics (the type of antibiotic was not reported) prior to admission. The sources of antibiotic treatment, i.e., self-treated or following a physicians‘ prescription, are unknown. For the comparison between qPCR negative and positive patients, there was no difference in median age between the two groups. The proportions of patients that were female, lived in a rural area, worked as farmers and did not use antimicrobial drugs before admission in the qPCR positive group were all statistically higher than those in the qPCR negative group (*p* = 0.000). In contrast, the proportion of patients admitted from the community in the qPCR positive group (54.8%) was lower than that in the qPCR negative group (68.3%), and this difference was also statistically significant (*p* = 0.000).

**Figure 2:**
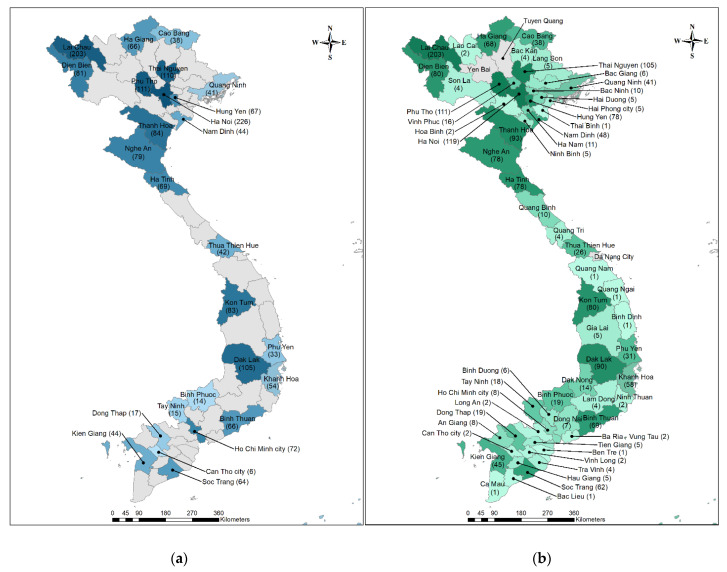
Frequency and Distribution of Enrolled Patients. (**a**) The number of patients that participated in the study by province of hospital. (**b**) The number of patients by province of residence. The color corresponds to patient distribution with the darker the color, the higher the number of enrollees.

The 1834 patients enrolled in the study originated from 60 of 63 provinces throughout the country. The distribution of patients by hospital varied considerably, though all hospitals applied the same inclusion criteria and procedures for recruitment (Figure 2a). While the National Hospital for Tropical Diseases (Hanoi) and Lai Chau Provincial Hospital (Lai Chau) recruited a large number of patients to the study, 226 and 203 respectively, Can Tho Central General Hospital (Can Tho) enrolled only six patients in total. The distribution of patients by province of residence shows that the largest numbers of patients came from the provinces of Lai Chau (203), Hanoi (119), Phu Tho (111) and Thai Nguyen (105) (Figure 2b). None of the enrolled patients lived in Tuyen Quang, Yen Bai and Da Nang city at the time of symptom onset.

**Figure 3:**
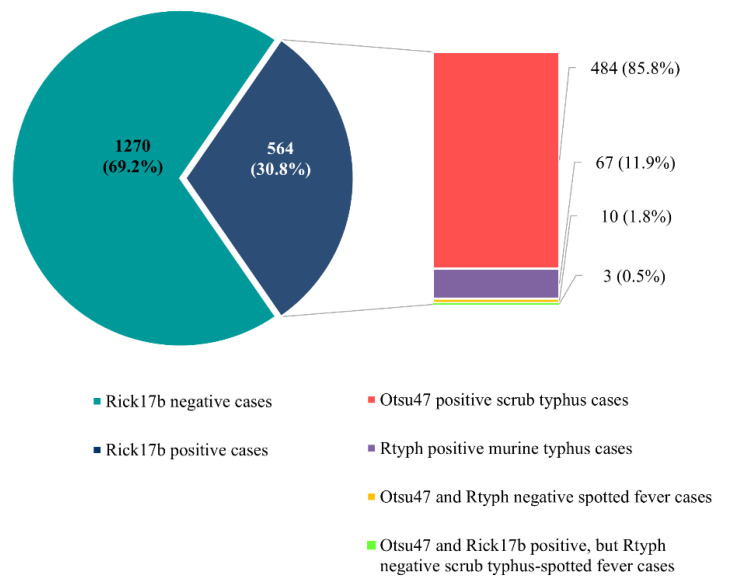
qPCR results for scrub typhus, murine typhus and spotted fever among 1834 febrile patients in the study.

Rickettsial DNA was detected in the PBMCs of 30.8% (564/1834) of enrolled patients. Of those, 11.9% (67) were positive by qPCR for *Rickettsia typhi*, and 85.8% (484) were positive by qPCR for *Orientia tsutsugamushi* (Figure 3). No samples were positive for *R. prowazekii*. Ten samples were positive using the *Rickettsia* genus-specific assay (Rick17b), but negative by either of the *R. typhi* or *R. prowazekii* assays. These Rick17b positive samples were subjected to multilocus sequence typing (MLST) targeting the conserved genes 17kDa antigen gene and *gltA* as well as the variable genes *ompA*, *ompB*, and *sca4* to determine the species of *Rickettsia* present in the samples. Due to low levels of the rickettsiae in the PMBCs, (Rick17b Ct values ranged from 35 to 38) amplicons were not obtained for any of the genes and the specific identify of the rickettsiae could not be determined. Given that the samples were positive for rickettsiae, but negative for any of the typhus group rickettsiae, the rickettsiae in these samples most likely belong to the spotted fever rickettsiae antigenic group. In addition, three (0.5%) patients were qPCR positive for both scrub typhus and rickettsiosis, with the following Otsu47 and Rick17b Ct values (34 and 36; 34 and 37; 34 and 36, respectively).

**Figure 4:**
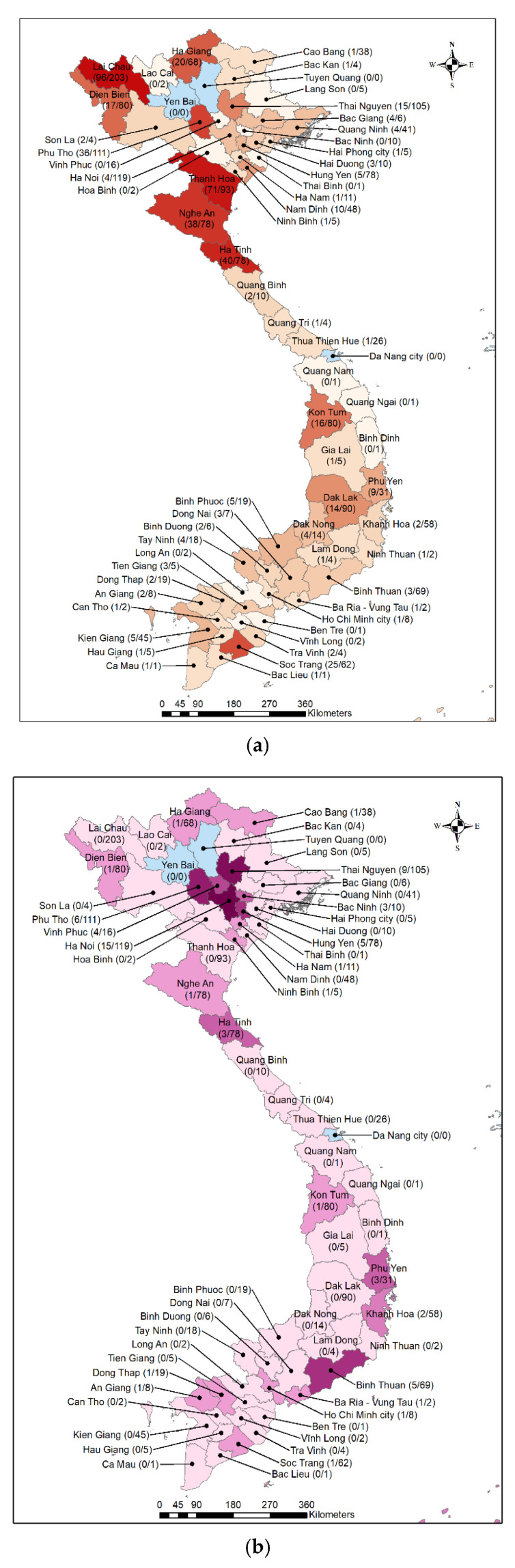

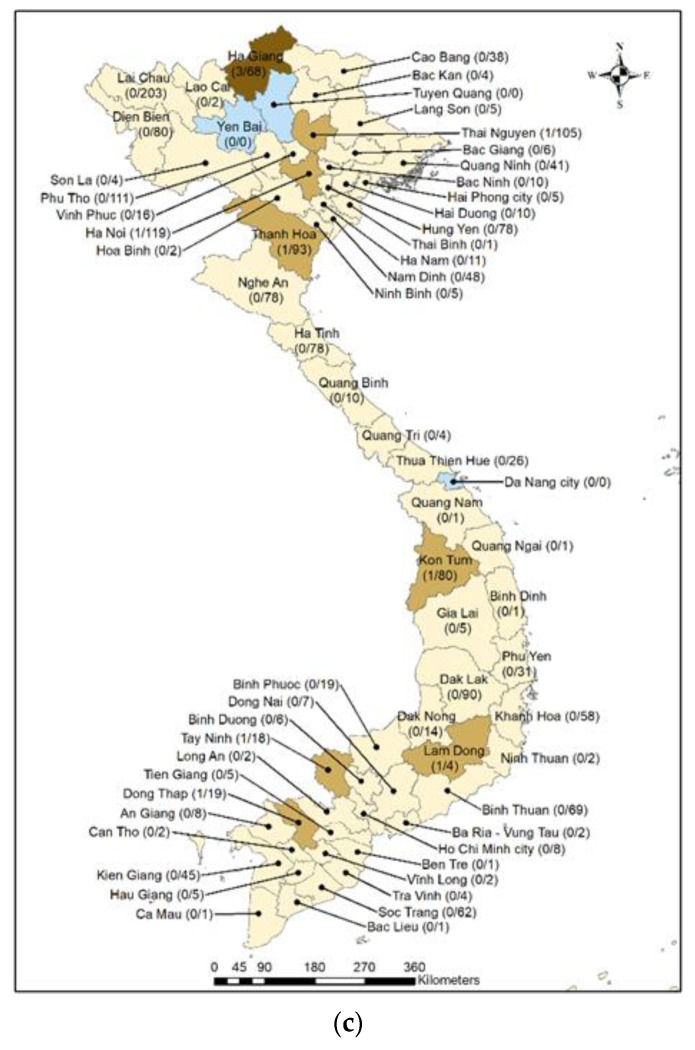
Distribution of confirmed cases of: (**a**) Scrub Typhus, *n* = 484; (**b**) Murine Typhus, *n* = 67; and (**c**) Spotted Fever, *n* = 10 (**c**) by provinces. The color density corresponds to the number of confirmed cases: the darker the color, the higher the number of confirmed cases. The fraction shows the number of confirmed case (numerator) and the number of recruited patients (denominator) for each province.

Confirmed cases of rickettsioses were distributed unevenly among 51 provinces in Northern, Central, and Southern Vietnam. The distribution map of 484 confirmed scrub typhus cases by province demonstrates that most of the cases originated from provinces in Northern and Central regions (Figure 4). The Northern provinces of Lai Chau, Thanh Hoa, Ha Tinh, and Nghe An ranked highest in terms of the number of confirmed cases. The largest proportion of cases was found in Thanh Hoa (71/93, 76.3%). In Southern Vietnam, with the exception of Soc Trang, which had 25 scrub typhus cases, the number of scrub typhus cases of the other provinces ranged from only one to five per province. Provinces without a participating hospital produced fewer study participants, however a proportion of those that did enroll were confirmed scrub typhus positive, even reaching 100% among those from Bac Lieu and Ca Mau. The 67 confirmed cases of murine typhus were sparsely distributed in 22 of 63 provinces across the country, but were found to be concentrated in Hanoi (15 cases) and neighboring provinces including Thai Nguyen (9 cases), Phu Tho (6 cases), Hung Yen (5 cases), Vinh Phuc (4 cases) and Bac Ninh (3 cases). The 10 cases of potential spotted fever rickettsiosis were even more sparsely distributed throughout the country, with 3 cases originating in Ha Giang and one case each in Hanoi, Thai Nguyen, Thanh Hoa, Kon Tum, Lam Dong, Tay Ninh and Dong Thap.

**Figure 5:**
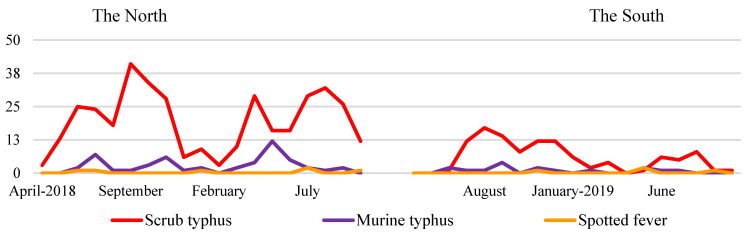
Frequency of Patients with Confirmed Scrub Typhus, Murine Typhus and Spotted Fever in the North and the South of Vietnam by Month.

There are two main climate regions in Vietnam, the North and the South, with the boundary, Hai Van Pass, between them [25]. The climate in the North includes four seasons: spring, summer, autumn, and winter; while the climate in the South has two clear seasons: dry and rainy. The frequency of patients with confirmed scrub typhus, murine typhus and spotted fever in the North and the South by month was assessed in order to evaluate the seasonality of rickettsial diseases (Figure 5). In the North, most scrub typhus patients were admitted to the hospitals in the summer and autumn months (June–October 2018 and April–August 2019). This was a period of high moisture and temperature in the North. Scrub typhus cases peaked in June and September 2018, and in April and August 2019 with 25 and 41, 29 and 32 cases, respectively. The number of murine typhus cases increased in July and November 2018, and in May 2019. In the South, scrub typhus cases were predominantly recorded in the rainy season in the months of July to December 2018 and Jun to August 2019. No seasonal trend for murine typhus was identified in the South. Cases of spotted fever occurred sporadically throughout the year in both regions.

Demographics, history, clinical manifestations, laboratory results, treatment therapy and outcomes of 549 confirmed cases of scrub typhus, murine typhus and spotted fever were assessed (Table 2). There was no difference in median age between scrub typhus and murine typhus groups, but there were significant differences in sex, occupation and living areas. Males (42, 63.6%) were more likely to be infected with murine typhus than females (24, 36.4%). Farming was the most common occupation reported among patients with scrub typhus (283, 58.5%), whereas only 18 (26.9%) patients with murine typhus were farmers.

The majority of scrub typhus cases were observed among rural (402, 83.1%) as opposed to urban residents. The proportions of patients transferred from other healthcare settings that received antimicrobial drug therapy before admission were not significantly different between the scrub typhus and murine typhus groups. The majority of patients in both groups were admitted to hospitals from their community: 264 (54.5%) for scrub typhus and 37 (55.2%) for murine typhus. For both scrub typhus and murine typhus cases, approximately 30% of patients used antibiotics before enrollment in our study.

At the time of enrollment in the study, the vast majority of scrub typhus and murine typhus patients presented with headaches, with 448 (92.6%) and 62 (92.5%), respectively. A considerably less common but still frequent symptom was myalgia, with 250 (51.7%) patients for scrub typhus and 44 (65.7%) for murine typhus. Other signs and symptoms of scrub typhus and murine typhus such as retro-orbital pain, sore throat, cough, nausea, vomiting, abdominal pain, diarrhea, and skin hyperemia were observed in no more than 50% of the patients in each group. Of these signs and symptoms there was a significant association between the disease type and the presence of myalgia/skin hyperemia (Table 2).

The most common physical signs among 484 confirmed scrub typhus patients were eschar (368, 76.0%), congested skin (273, 56.4%), lymphadenopathy (243, 50.2%) and conjunctivitis (163, 33.7%) (Table 2). Eschars were found on different parts of the body, but most commonly in the axillary region (69, 19.1%), inguinal area (52, 14.4%) and genitals (38, 10.5%). Liver and spleen enlargement were reported in only 28 (5.8%) and 31 (6.4%) scrub typhus patients, respectively. Congested skin, conjunctivitis, and rash in comparison with the other signs including eschar, lymphadenopathy, liver and spleen enlargement were observed among a minority of scrub typhus patients. These observations were less frequently observed among murine typhus than scrub typhus patients (*p* < 0.05) (Table 2). Common physical signs and symptoms among murine typhus cases included congested skin (54, 80.6%) and conjunctivitis (36, 53.7%). Eschars were reported in four patients.

Upon admission to the hospital, 417 and 376 of 1834 study patients were tested for complete blood count and biochemical markers, respectively. In those tested, the mean red blood cell counts were significantly lower in patients with murine typhus than in those with scrub typhus (*p* = 0.027). Platelet counts of less than 100 G/l were observed for approximately half of patients in both groups. Elevated liver aminotransferases were reported in more than 85% of scrub typhus and murine typhus patients. There was a significant association between the groups of rickettsial diseases and elevated alanine aminotransferase/blood albumin levels (Table 2).

Of the 561 patients with scrub typhus, murine typhus and spotted fever, 557 received the antimicrobial therapy for treatment of their current conditions. Of these, 548 (97.7%) were provided the treatment of choice for rickettsiosis including doxycycline, chloramphenicol and azithromycin (Table 2). Among four murine typhus patients who had an eschar, three received doxycycline for treatment and the other was given non-protocol antibiotics including ciprofloxacin, cefotaxime, vancomycin, ceftriaxone, and amikacin. A minority of patients required respiratory support (35, 6.2%), albumin transfusion (26, 4.7%) or dialysis (3, 0.5%), and no statistical differences were observed between scrub and murine typhus groups. The majority of patients recovered well after treatment, with 87.0% (474) of patients afebrile <3 days after treatment began. Scrub typhus patients responded to treatment better than murine typhus cases (*p* = 0.024). The median hospital stay for scrub typhus and murine typhus were six (range of five to eight) and seven (range of five to eight) days, respectively.

Six scrub typhus and one murine typhus patient suffered from shock. Six deaths were recorded, of which five were patients with scrub typhus and one with murine typhus. These patients died at home after being discharged from the hospital at the request of their families. We observed four murine typhus patients that were treated with azithromycin and all recovered well after treatment.

The median age of patients with spotted fever group rickettsiosis, 28 (IQR: 18–37), was lower than the other groups. Almost all patients infected with SFGR were male (eight, 80%) and lived in rural areas (six, 60%). Farming was the occupation of 50% of spotted fever patients. While five patients were admitted to the hospitals from their community, the other five were transferred from other hospitals or clinics. There were many symptoms and physical signs presented in scrub typhus and murine typhus patients that were absent from spotted fever rickettsiosis patients such as vomiting, abdominal pain, diarrhea, skin hyperemia, subcutaneous hemorrhage, mucous membrane hemorrhage, and rales. An eschar was observed in one patient with spotted fever rickettsiosis. Abnormal findings in blood test such as total bilirubin >17 µmol/L, albumin <32 g/L, and creatinine >120 µmol/L were not reported in these cases. Eight of the 10 spotted fever patients were treated with a drug of choice in the treatment of rickettsiosis (doxycycline, chloramphenicol, or azithromycin), and one received non-protocol antibiotic therapy. There were no deaths or cases of shock among spotted fever rickettsiosis patients.

## 4. Discussion

This is the first comprehensive picture of the prevalence and distribution of rickettsial diseases throughout Vietnam. The results of this study not only build upon the existing knowledge of physicians regarding the epidemiology of rickettsial diseases, but will also improve physicians’ understanding of clinical presentations and the diagnosis of rickettsial diseases. Enhancing the understanding of rickettsial diseases will improve the early diagnosis and appropriate treatment of these under-recognized, potentially life-threatening, but treatable diseases.

In this study, a total of 1834 patients from five national and 22 provincial hospitals of the 26 provinces participated. Importantly, the home residences of these patients represented all provinces with the exception of Yen Bai, Tuyen Quang and Da Nang, providing incredible geographic distribution. The 5 national hospitals in the study received transfer patients from provincial hospitals not included in the study, thereby increasing the breadth of reach of the study. For example, the National Hospital for Tropical Diseases (Hanoi) recruited 226 patients to the study in total but almost half of these patients originated from other provinces in the North (Figure 2).

Of the 1834 patients with clinically suspected rickettsiosis recruited in this study, 564 patients were confirmed to be positive for a rickettsial disease, including 484 with scrub typhus, 67 with murine typhus and 10 with spotted fever (Table 2). These results showed that scrub typhus was the most common rickettsial infection among hospitalized, undifferentiated febrile patients, followed by murine typhus and spotted fever. Scrub typhus, a mite-borne disease caused by *O. tsutsugamushi*, has been previously reported to be endemic within the so-called Tsutsugamushi Triangle, an area covering Asia, Australia and islands in the Indian and Pacific Oceans [26]. However, recently the endemic area of scrub typhus was no longer limited within the Tsutsugamushi Triangle but has been found outside of the area and is related to the new *Orientia* species [27]. In Southeast Asia, including Vietnam, scrub typhus has been reported as the most common and important rickettsiosis among acute undifferentiated or non-malarial/dengue fever cases [9,10,28,29,30]. The finding in this study of the high prevalence of scrub typhus among the study population is in line with the observations of the above research. Murine typhus, a flea-borne disease caused by *R. typhi*, has been shown to be an important consideration for the cause of nonspecific febrile illnesses [31] but it was less frequently found among patients with undifferentiated fever than scrub typhus. In two recent studies from the North and Central regions of Vietnam, murine typhus was reported in 3.3% and 4.8% of patients with acute undifferentiated fever, respectively [9,11]. The proportion of patients with murine typhus in this study population was similar to those reports. This is one of a few studies reporting the presence of spotted fever in Vietnam. However, the specific rickettsial agents causing the spotted fever were not identified due to the lack of sufficient clinical sample material.

Based on geographical features, Vietnam is divided into three main regions known as Northern, Central and Southern. While previous studies primarily reported the presence of the diseases in Northern and Central Vietnam [9,11,16,17,18,32], this study provides evidence of the widespread prevalence of rickettsioses in all three regions of the country. We found that 564 rickettsial cases originated from 51 out of 63 provinces throughout Vietnam. The majority of scrub typhus patients came from Northern and Central provinces such as Lai Chau, Phu Tho, Thanh Hoa, Nghe An and Ha Tinh. Notably, in the Southern region among 19 provinces, the low number of scrub typhus patients recorded included Long An, Vinh Long and Ben Tre with no cases and 15 others that reported typhus cases, with the numbers ranging from two to five. One exception to the low occurrence of scrub typhus in the Southern region was Soc Trang province, which had 25 cases.

For both murine typhus and spotted fever, it was observed that the Northern region had higher numbers of cases in comparison with the Central and Southern regions. In particular, murine typhus cases were mainly distributed in Hanoi and its adjacent provinces, and five of the 10 spotted fever cases were observed in three Northern provinces. Scattered cases of murine typhus and spotted fever were recorded in 8 Central and 5 Southern provinces, respectively. These results shine a light on the inadequacies and limitations in research of rickettsial diseases in Vietnam, especially the Southern region. Although scrub typhus and murine typhus were reported early in Saigon (now Ho Chi Minh city) during and before the 1970s [12,13,14,15] and most recently in 2003 [33], research on rickettsial diseases in the North was neglected for most of the last 20 years.

One limitation of this study is with regard to the distribution of participating hospitals and how that affected the geographic distribution of patients enrolled in the study. Patients from provinces with a participating hospital are over represented in the study based solely on proximity and not necessarily due to an increased risk of rickettsial diseases relative to provinces without a participating hospital. Future studies are needed to fully understand the prevalence and risk of rickettsial diseases throughout the country. Another limitation of the study was the use of non-protocol antibiotics among nine patients due to the temporary lack of hospital supply.

We generally found that rickettsial diseases peaked during the summer and autumn in the Northern region and the rainy season in the Southern region (Figure 5). Both the summer and autumn periods were characterized by hot and humid seasons for both regions. In these periods, ectoparasites would be more active and farmers spend more time in the fields. Previous studies have described the seasonality of rickettsial diseases similar to our findings [9,16].

When comparing the demographics, history and clinical manifestations of 484 scrub typhus and 67 murine typhus patients, we found statistically significant differences in sex, occupation, living areas and most physical signs between the two groups. This finding differs from what we previously described in a study of 302 clinically suspected rickettsiosis cases admitted to two large referral hospitals in Hanoi [9]. According to that study, the differences were significant in the distribution by sex and the presence of eschar only. Eschars are recognized as typical clinical manifestations and are used to aid in the diagnosis of scrub typhus and other mite- or tick-borne rickettsioses, with the specificity for scrub typhus reaching up to 98.9% [34]. However, the occurrence of eschars in patients with scrub typhus is highly variable and has been reported to range from seven to about 90% in various studies [35,36]. The prevalence of eschars among scrub typhus cases in this study was 76.0%, which is higher than reported in other domestic studies [9,11,16,32] but still within the published range. Interestingly, study physicians observed eschars in four patients with murine typhus. Eschars are not known to be associated with murine typhus due to its specific transmission route. An inoculation lesion has been recorded in one case of murine typhus but it was not an eschar [37]. The occurrence of eschars in four murine typhus patients may have resulted from the study physicians mistakenly identifying a non-eschar lesion as an eschar. It is possible that physicians might have been identifying bites or lesions as eschars and have been giving murine typhus patients a diagnosis of scrub typhus. Additional education of healthcare providers in the identification of eschars may be of benefit to clinicians in Vietnam where scrub typhus is common. Fortunately, the possible mistake of identifying an eschar among four murine typhus patients did not affect the patients’ outcome since scrub and murine typhus patients received the same antibiotic therapy.

Most study patients were treated with doxycycline, chloramphenicol or azithromycin, and recovered well in the first three days of treatment. Although a study by Paul N. Newton in 2019 indicated that azithromycin was not as good as doxycycline in the treatment of murine typhus, four murine typhus patients that received azithromycin in our study responded well to treatment [38]. Previous studies have demonstrated that early diagnosis and prompt initiation of proper antibiotic treatment in rickettsial illnesses often results in recovery [39,40]. However, the proportion of afebrile scrub typhus patients ≤72 h after treatment began (418, 88.2%) was significantly higher than that of patients with murine typhus (47, 77.1%). A systematic review indicated that the mortality of untreated scrub typhus is around 6%, but ranges from 0 to 70% (40). The fatality rates of scrub typhus and murine typhus in our study were 1.1% and 1.5%, respectively. Previous studies have reported no fatal cases of murine typhus, with fatality rates of 0.4–4.9% for scrub typhus [9,16].

Individuals with spotted fever cases tended to be quite young, with 75% being under 37 years of age. This finding contributed to the assumption in previous studies that spotted fevers most often affect children and young adults [41,42,43]. Like other rickettsioses, the clinical presentations of spotted fevers can vary widely, and an eschar occasionally occurs at the site of the tick or mite bite [43]. In our study, the absence of some symptoms and signs among spotted fever cases in comparison with scrub typhus and murine typhus might be caused by the small number observed (10 cases). We found that the proportion of patients with eschars was only 10% (1/10). However, it has been shown that the presence or absence of an eschar in patients with spotted fevers was highly variable and different among pathogenic SFGR [44].

In conclusion, rickettsial diseases are widely distributed in all regions of Vietnam. The presentations of rickettsial diseases were often nonspecific and easy to be misdiagnosed with other febrile infections. Awareness about both epidemiology and the clinical manifestations of pathogens in a physician’s field of practice is important for the timely diagnosis and proper treatment of rickettsial diseases, especially in cases where there is a lack of proper diagnostic tests.

## Figures and Tables

**Table 1:** General characteristics of patients enrolled in the study.

Characteristics		Total	qPCR Negative	qPCR Positive	*p*-Value †
		**Median (p25–p75)**			
Age (*n* = 1834)		39 (27–55)	38 (27–54)	42 (24–58)	0.2883 ‡
		**n (%)**			
Sex (*n* = 1831)	Female	861 (47.0)	554 (44.1)	307 (53.4)	**0.000**
	Male	970 (53.0)	702 (55.9)	268 (46.6)	
Residence (*n* = 1832)	Rural area	1271 (69.4)	813 (64.8)	458 (79.2)	**0.000**
	Urban area	561 (30.6)	441 (35.2)	120 (20.8)	
Occupation (*n* = 1843)	Farmer	866 (47.2)	550 (43.8)	316 (54.7)	**0.000**
	Non-farmer	968 (52.8)	706 (56.2)	262 (45.3)	
Origin (*n* = 1834)	Admitted from community	1175 (64.1)	858 (68.3)	317 (54.8)	**0.000**
	Transferred from another hospital or clinic	659 (35.9)	398 (31.7)	261 (45.2)	
Used antimicrobial drugs before admission (*n* = 1834)	Yes	426 (23.2)	264 (21.0)	162 (28.0)	**0.001**
	No	1408 (76.8)	992 (79.0)	416 (72.0)	

† *p* value obtained using Fisher exact test, except where noted. ‡ *p* value obtained using Mann-Whitney test.

**Table 2:** Demographics, history, clinical manifestations, laboratory results, treatment, and outcomes of 484 scrub typhus, 67 murine typhus and 10 spotted fever patients.

Characteristics	Scrub Typhus *n* = 484	Murine Typhus *n* = 67	Spotted Fever *n* = 10	Total *n* = 561	*p* Value ^§^
**Demographics**					
Age, median (IQR) (*n* = 560)	43 (24–58)	43 (31–54)	28 (18–37)	42.5 (24–58)	0.344 ^¶^
Male, (*n*, (%)), *n* = 560)	206 (42.6)	42 (63.6)	8 (80.0)	256 (45.7)	**0.001**
Female (*n*, (%), *n* = 560)	278 (57.4)	24 (36.4)	2 (20.0)	304 (54.3)	
Farmer, *n* = 561	283 (58.5)	18 (26.9)	5 (50.0)	306 (54.6)	**0.000**
Residence in rural area, *n* = 561	402 (83.1)	36 (53.7)	6 (60.0)	444 (79.1)	**0.000**
**History**					
Admitted from community, *n* = 561	264 (54.5)	37 (55.2)	5 (50.0)	398 (54.6)	1.000
Transferred from other hospital or clinic, *n* = 561	220 (45.6)	30 (44.8)	5 (50.0)	255 (45.4)	
Received antimicrobial drugs before admission, *n* = 561	135 (27.9)	20 (29.9)	3 (30.0)	158 (28.2)	0.772
**Symptoms**					
Headache, *n* = 561	448 (92.6)	62 (92.5)	10 (100)	520 (92.7)	1.000
Myalgia, *n* = 561	250 (51.7)	44 (65.7)	9 (90.0)	303 (54.0)	**0.036**
Retro-orbital pain, *n* = 560	105 (21.7)	21 (31.3)	2 (20.0)	128 (22.9)	0.088
Sore throat, *n* = 561	117 (24.2)	24 (35.8)	3 (30.0)	144 (25.7)	0.098
Cough, *n* = 560	220 (45.6)	34 (50.8)	5 (50.0)	259 (46.3)	0.435
Nausea, *n* = 561	147 (30.4)	23 (34.3)	4 (40.0)	174 (31.0)	0.573
Vomiting, *n* = 561	72 (14.9)	11 (16.4)	0	83 (14.8)	0.717
Abdominal pain, *n* = 561	95 (19.6)	7 (10.5)	0	102 (18.2)	0.092
Diarrhea, *n* = 560	59 (12.2)	6 (9.0)	0	65 (11.6)	0.547
Skin hyperemia, *n* = 560	124 (25.7)	27 (40.3)	0	151 (27.0)	**0.040**
**Physical signs**					
Congested skin, *n* = 561	273 (56.4)	54 (80.6)	4 (40.0)	331 (59.0)	**0.000**
Conjunctivitis, *n* = 551	163 (33.7)	36 (53.7)	3 (10.0)	202 (36.0)	**0.002**
Eschar, *n* = 561	368 (76.0)	4 (6.0)	1 (10.0)	373 (66.5)	**0.000**
Rash, *n* = 561	118 (24.4)	26 (38.8)	0	144 (25.7)	**0.017**
Lymphadenopathy, *n* = 561	243 (50.2)	7 (10.5)	1 (10.0)	251 (44.7)	**0.000**
Liver enlargement, *n* = 561	28 (5.8)	0	2 (20.0)	30 (5.4)	**0.037**
Spleen enlargement, *n* = 561	31 (6.4)	0	1 (10.0)	32 (5.7)	**0.024**
Subcutaneous hemorrhage, *n* = 560	13 (2.7)	5 (7.5)	0	18 (3.2)	0.056
Mucous membrane hemorrhage, *n* = 561	5 (1.0)	2 (3.0)	0	7 (1.3)	0.205
Rales, *n* = 561	64 (13.2)	8 (11.9)	0	72 (12.8)	1.000
**Laboratory test results at admission**					
Erythrocytes, T/L, median (IQR), *n* = 417	4.4 (3.9–4.7)	4.5 (4.2–4.9)	4.4 (4.1–4.8)	4.4 (4.0–4.7)	**0.027** ^¶^
Leukocytes, T/L, median (IQR), *n* = 417	7.5 (5.6–10.3)	6.5 (5.0–8.7)	6.2 (4.2–7.7)	7.4 (5.5–10.0)	**0.049** ^¶^
Platelets, G/L, median (IQR), *n* = 415	100 (66–144)	87.5 (65–133)	177.5 (75–269.5)	100 (66–144)	0.303 ^¶^
Platelet < 100 G/L, *n* = 415	187 (51.5)	20 (45.5)	6 (75.0)	213 (51.3)	0.524
Alanine aminotransferase > 40 IU/L, *n* = 376	282 (85.2)	36 (97.3)	7 (87.5)	325 (86.4)	**0.042**
Aspartate aminotransferase ST > 37 IU/L, *n* = 375	316 (95.8)	35 (94.6)	8 (100)	359 (95.7)	0.670
Total bilirubin > 17 µmol/L, *n* = 82	18 (23.4)	2 (18.2)	0	20 (24.4)	1.000
Albumin < 32 g/L, *n* =111	54 (54.6)	1 (8.3)	0	55 (49.6)	**0.004**
Creatinine > 120 µmol/L, *n* = 352	19 (6.1)	1 (3.0)	0	20 (5.7)	0.706
**Treatment**					
One of three protocol-specific antibiotics (Doxycycline, Chloramphenicol and Azithromycin), *n* = 661	478 (98.8)	62 (92.5)	8 (80.0)	548 (97.7)	-
Non-protocol antibiotics, *n* = 561	4 (0.8)	4 (6.0)	1 (10.0)	9 (1.6)	
Other therapies, (*n* = 561)	2 (0.4)	1 (1.5)	1 (10.0)	4 (0.7)	
Respiratory support, *n* = 561	31 (6.4)	4 (6.0)	0	35 (6.2)	1.000
Albumin transfusion, *n* = 557	23 (4.8)	3 (4.6)	0	26 (4.7)	1.000
Blood purification, *n* = 555	3 (0.6)	0	0	3 (0.5)	1.000
**Outcomes**					
Death, *n* = 561	5 (1.0)	1 (1.5)	0	6 (1.1)	0.542
No. days in hospital, *n* = 555	6 (5–8)	7 (5–8)	7 (5–7)	6 (5–8)	0.128 ^¶^
Suffering from shock, *n* = 549	6 (1.2)	1 (1.5)	0	7 (1.3)	0.595
Afebrile ≤ 72 h after treatment began, *n* = 545	418 (88.2)	47 (77.1)	9 (90.0)	474 (87.0)	**0.024**

^§^*p* value obtained when comparing between scrub and murine typhus groups, using Fisher’s exact test, except where noted. ^¶^ *p* value obtained when comparing between scrub and murine typhus groups using Mann-Whitney test.

## Data Availability

Not applicable.
